# Carnrick’s Soluble Food

**Published:** 1886-08

**Authors:** 


### *CARNRICK'S SOLUBLE FOOD.*

Dr. C. F. Denny, in a communication to the editor of *Northwestern Lancet*, says:

Not long since I had brought to me a child of six months, suffering from the following symptoms:

Constipation at times, irregular action of bowls, regurgitation of food, and an asthmatic cough. Its mouth was full of thrush sores, and its appearance one of poor nourishment.

It had been given a number of infants' foods in vain, one of which I prescribed myself.

By means of mild medication, directed towards the cough and stomach, something was accomplished. Finally I gave "CARNRICK'S SOLUBLE FOOD," and had the satisfaction of having it retained, and, at last accounts, the child was doing nicely.

I am inclined to think this food is worthy of attention on the part of the profession.

It recommends itself in that it contains caseine, rendered soluble by pancreatine, starch converted into dextrine and maltose; hence, it requires but little preparation, and that is so simple, mistakes cannot occur.

It requires no addition of milk.

It has the advantages, and none of the disadvantages, of the many foods now in the market, and forms a nearly physiological substitute for mother's milk.

